# Bone morphological feature extraction for customized bone plate design

**DOI:** 10.1038/s41598-021-94924-9

**Published:** 2021-08-02

**Authors:** Lin Wang, Kaijin Guo, Kunjin He, Hong Zhu

**Affiliations:** 1 School of Medical Information and Engineering, Xuzhou Medical University, Xuzhou, 221004 People’s Republic of China; 2 Department of Orthopedics, Affiliated Hospital of Xuzhou Medical University, Xuzhou, 221006 People’s Republic of China; 3 College of Internet of Things Engineering, Hohai University, Changzhou, 213022 People’s Republic of China

**Keywords:** Bone, Mechanical engineering

## Abstract

Fractures are difficult to treat because of individual differences in bone morphology and fracture types. Compared to serialized bone plates, the use of customized plates significantly improves the fracture healing process. However, designing custom plates often requires the extraction of skeletal morphology, which is a complex and time-consuming procedure. This study proposes a method for extracting bone morphological features to facilitate customized plate designs. The customized plate design involves three major steps: extracting the morphological features of the bone, representing the undersurface features of the plate, and constructing the customized plate. Among these steps, constructing the undersurface feature involves integrating a group of bone features with different anatomical morphologies into a semantic feature parameter set of the plate feature. The undersurface feature encapsulates the plate and bone features into a highly cohesive generic feature and then establishes an internal correlation between the plate and bone features. Using the femoral plate as an example, we further examined the validity and feasibility of the proposed method. The experimental results demonstrate that the proposed method improves the convenience of redesign through the intuitive editing of semantic parameters. In addition, the proposed method significantly improves the design efficiency and reduces the required design time.

## Introduction

Fractures caused by trauma are common, where a large force and a short action time result in severe damage to the body^1^. The most common types of implants are orthopedic plates, whose main function is maintaining the reduction state of the fracture end and control the length, axis, and rotation of the diaphysis to provide good stability^2^. This helps to shorten the patient’s healing time. In recent years, the demand for orthopedic plates has increased significantly for multiple reasons, including continuous improvements in medical treatment, health awareness, and the existence of an aging population. Clinical results show that bone plates that are well matched with the shape of the bone surface can -assist the surgeon in reducing the fracture fragment with the help of the plate, provide good stability, and reduce the impact of the bone plate on soft tissue^3,4^. Designing a poorly matched plate can lead to surgical failure and other problems^5^. Clinically, mismatching of the bone plate and the patient’s bone often causes surgery failure^6,7^. As a result, the plate must be precisely shaped to fit the bone shape. Customized plates constructed according to specific anatomical shapes and fracture types improve the process of fracture healing and are considered next-generation medical devices. A computational design process for anatomical enhancement of plates was introduced, which can help manufacturers in identifying anatomically compliant implant designs in the early phases of product development^8^. However, patients have significantly different anatomical structures and behavioral movements^9,10^, making it difficult to design plates for individual patients that conform to their anatomical characteristics and conditions^11^.

In the traditional design process of a customized plate, orthopedic surgeons and designers need to communicate repeatedly, complicating the entire process^12,13^. Recently, researchers in China and other countries have proposed various design methods. The 3D-surface model of a detailed femur and the corresponding fixation plate were represented with high-level feature parameters, and the shape of the specific plate was recursively modified to obtain the optimal plate for a specific patient^14^. In reference 15^15^, the researchers constructed a custom-designed plate with semantic parameters, based on an average bone model created from existing bone models, promoting the quality and efficiency of orthopedic plate design. However, that study only considered femoral feature parameters as parameter constraints when constructing the surface model of the fractured femur. The influence of the anatomical morphological features of the femur on the feature parameters of the bone plate was not explored in detail in the above methods. However, excavation of the anatomical features of the bone is key to achieving a high match between the bone plate and the bone surface. Extracting bone morphological features is essential to designing orthopedic plates. Converting the conventional plate modeling content into a feature model that is constructed according to bone features is expected to be a more efficient and simpler design process.

Feature extraction—a common data-processing technique in machine learning—changes the dimensions of the feature space^16^. Generally, feature extraction involves collecting a large set of parameters and using a typical feature extraction method, such as a decision tree^17^ or deep convolutional neural network^18,19^, to extract useful information. Principal component analysis (PCA), a common feature extraction method, has a wide range of orthopedic applications. A PCA model helped predict the shapes of the pelvis and femur from palpable anatomical landmarks^20^. The PCA method also helped establish the relationship between the external body shape and the internal skeleton of the upper body^21^. Khan et al.^22^ used PCA to report the growth and degenerative patterns of the human spine. Some noticeable lumbar spine features (e.g., vertebral height, disc height, disc signal intensity, paraspinal muscle, subcutaneous fat, psoas muscle, and cerebrospinal fluid) were used to examine the variations in the lumbar spine with natural aging. In our previous work^23^, we used the factor analysis method to extract “size factor” and “angle factor” and noticed that it was related to the person’s height. Extraction of bone morphological features helps to obtain useful information from high-dimensional bone morphological data to reduce its redundancy^24–26^. To facilitate the design of orthopedic plates, this study expanded the number of femur parameters containing the proximal, shaft, and distal regions. Subsequently, we used PCA to extract several important principal components, which were then used to establish the plate feature.

This study aimed to provide an efficient and convenient approach for designing plate features based on bone features. Hence, two major requirements should be satisfied:

- The plate features must be constructed according to anatomical morphological information.
- The feature parameters of the plate must be stratified to improve the adjustment efficiency.

To satisfy these two requirements, we propose an effective method for extracting bone morphological features and guiding a customized plate design.

The remainder of this paper is organized as follows. In “Overview” section provides a brief overview of the proposed method. The main steps of the proposed method (i.e., extracting the bone morphological features, constructing the plate feature model, and generating the customized plate) are presented in “Extraction of bone morphological features”, “Construction of the plate feature model”, and “Generation of the customized plate” sections, respectively. Finally, the conclusions are presented in “Discussion and conclusion” section.

## Overview

**Figure 1:**
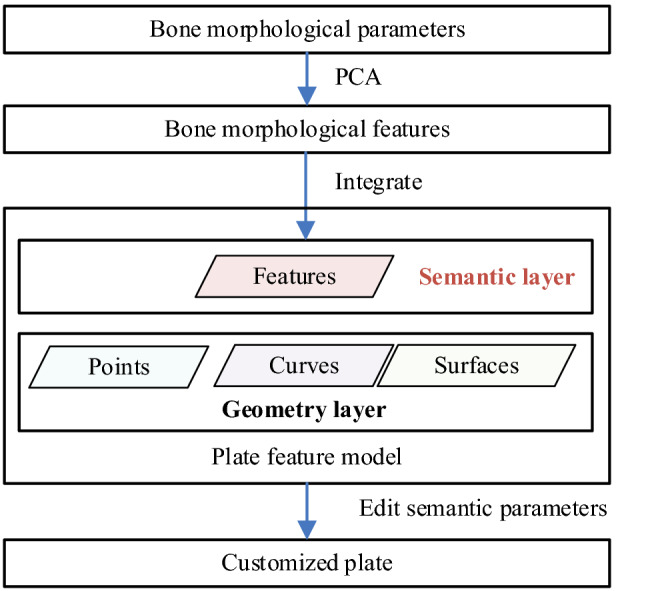
Workflow of the proposed method.

Figure 1 shows the extracted bone morphological features which were integrated into the plate feature model in this study. The steps for this method are as follows:

1. We used PCA to extract bone morphological features.
2. We integrated bone morphological features into the semantic layer of the plate feature model.
3. We constructed a customized plate by editing the semantic parameters defined in the plate feature model according to the bone morphological parameters of an individual patient.

This study uses two types of features from the above steps:

- Bone features, including features of the fracture and its morphological features, which consist of the following aspects. Fracture features are an important basis for judging fracture types, which are usually characterized by imaging fracture lines^27^. Morphological features describe the anatomical structure of the bone, which is usually extracted from parameters using a semantic form^28^. After confirming the fracture type of the patient, we only focused on bone morphology.
- The plate feature includes a group of geometric shapes that embody the designer’s intention and constitutes a certain set of engineering semantics^29^. In this way, the plate feature supports editing the plate in the semantic feature layer and avoids tedious operation on the geometric layer at the bottom.

## Extraction of bone morphological features

### Bone sample data collection for PCA

This study focused on 100 right femurs of unrelated healthy adults who belonged to the Chinese Han ethnic group, with an average age of 47 years. The parameter values of 100 femur samples (expressed as mean ± standard deviation, where length is expressed in mm and angle in °) are as follows:

- Parameters of the proximal femur include the vertical height of the femoral head (*H*_fh_, 47.95±5.33), collodiaphyseal angle (*A*_fn_, 126.18±6.12), eccentric distance (*L*_fhs_, 38.75±6.25), neck length (*L*_fn_, 50.80±6.19), femoral head diameter (*D*_fh_, 43.33±3.40), femoral neck diameter (*D*_fn_, 33.08±3.25), length of great trochanter (*L*_t_, 65.86±4.03), bump height of great trochanter (*H*_t1_, 10.73±1.09), and interior offset of great trochanter (*H*_t2_, 17.57±2.36). These parameters are denoted in vector form as *X*_*i*_ (*i* = 1, 2, 3, …, 9), in the same order presented above after normalization.
- Parameters of the femoral shaft include the femoral shaft coronal diameter (*D*_fs_, 25.94±2.23), length of the femoral shaft (*H*_fs_, 269.19±16.64), and femoral shaft bending angle (*A*_fs_, 173.39±1.51).
- Parameters of the distal femur also include distal transverse diameter (*L*_df_, 75.10±6.00), anterior and posterior length of medial condyle (*L*_m_, 56.03±3.74), anterior and posterior length of external condyle (*L*_l_, 59.95±4.00), length of anterior condyle line (*L*_a_, 32.77±2.53), length of posterior condyle line (*L*_p_, 52.06±4.05), height of medial condyle (*H*_m_, 55.35±3.71), height of lateral condyle (*H*_l_, 59.73±3.98), angle of medial condyle (*A*_m_, 81.33±1.87), angle of lateral condyle (*A*_l_, 85.60±1.97), angle of the anterior condyle (*A*_a_, 8.25±2.85), angle of posterior condyle (*A*_p_, 4.13±1.63), angle of trochlear groove (*A*_s_, 134.11±4.19), depth of trochlear groove (*H*_tg_, 6.04±0.75), the height difference between the medial and lateral condyle (*H*_ml_, 4.37±0.51), rate of femoral surface (*C*_f_, 1.25±0.09), and joint inclination of medial and lateral condyle (*A*_ml_, 12.98±2.86). Similarly, these parameters are denoted in vector form, *Y*_*i*_ (*i* = 1, 2, 3, …, 16), in the same order as presented above after normalization.

To ensure that the collected data represent the general population and avoid severe deviations, the samples included in the analysis should be close to a normal distribution. The height of individuals follows a normal distribution, and the femoral length is often proportional to the height; therefore, we can reasonably assume that the femoral length should also follow a normal distribution^30^. Experiments with 100 samples show that the femoral length distribution is left skewed, with a skewness value of − 0.07, mean of 420.072 mm, and standard deviation of 22.968 mm. Notably, the deviation degree is small, and the collected samples can be considered as being reasonably representative of the general population. Furthermore, we used the Kaiser–Meyer–Olkin (KMO) test31 to compare simple and partial correlation coefficients between the variables. The KMO statistic was between 0 and 1. The closer the KMO value is to 1, the stronger the correlation between variables, and the more suitable the original variables for PCA. In the present study, the KMO statistic was 0.731. Furthermore, Bartlett’s sphericity test^31^, which can also test the correlation between variables, indicates a significance value (i.e., Sig.) of 0.00, which is less than 0.05. These results indicate that the collected set of samples is particularly suitable for PCA requirements.

### Extraction of the principal components

PCA is the most commonly used linear dimensionality-reduction method. It maps the high-dimensional data to a low-dimensional space via linear projection and extracts most information in the dimensionality (maximum variance) of the projected data to use fewer data dimensions while retaining as much feature information as possible from the original data. By mapping an *n*-dimensional feature to a *k*-dimensional feature, in which *k* is often less than *n*, the *k*-dimensional feature is reconstructed, which is called the principal component^31^. In this way, we can use the principal components to reflect the information of the original variable and replace the original variable for an in-depth study. The steps for feature extraction via PCA are as follows.

1. We calculated the covariance matrix, eigenvalues, and eigenvectors. The selection principles of the *k* eigenvectors are as follows:
  The eigenvalue must be greater than 0.5, and ideally greater than 1.
  The cumulative contribution rate should be greater than 90%^31^.
2. We sort the eigenvalues and retain the eigenvectors corresponding to the first k largest eigenvalues.
3. We converted the original features into a new space constructed by the *k* feature vectors obtained above.

Notably, the KMO statistic for parameters of the femoral shaft is 0.465, which is much lower than the average level of 0.731, suggesting that the correlation between the parameters of the femoral shaft is relatively small. Furthermore, there were fewer femoral shaft parameters. Therefore, extracting the principal components of the femoral shaft parameters is not required. Feature extraction of the proximal and distal femurs is described below.

**Figure 2:**
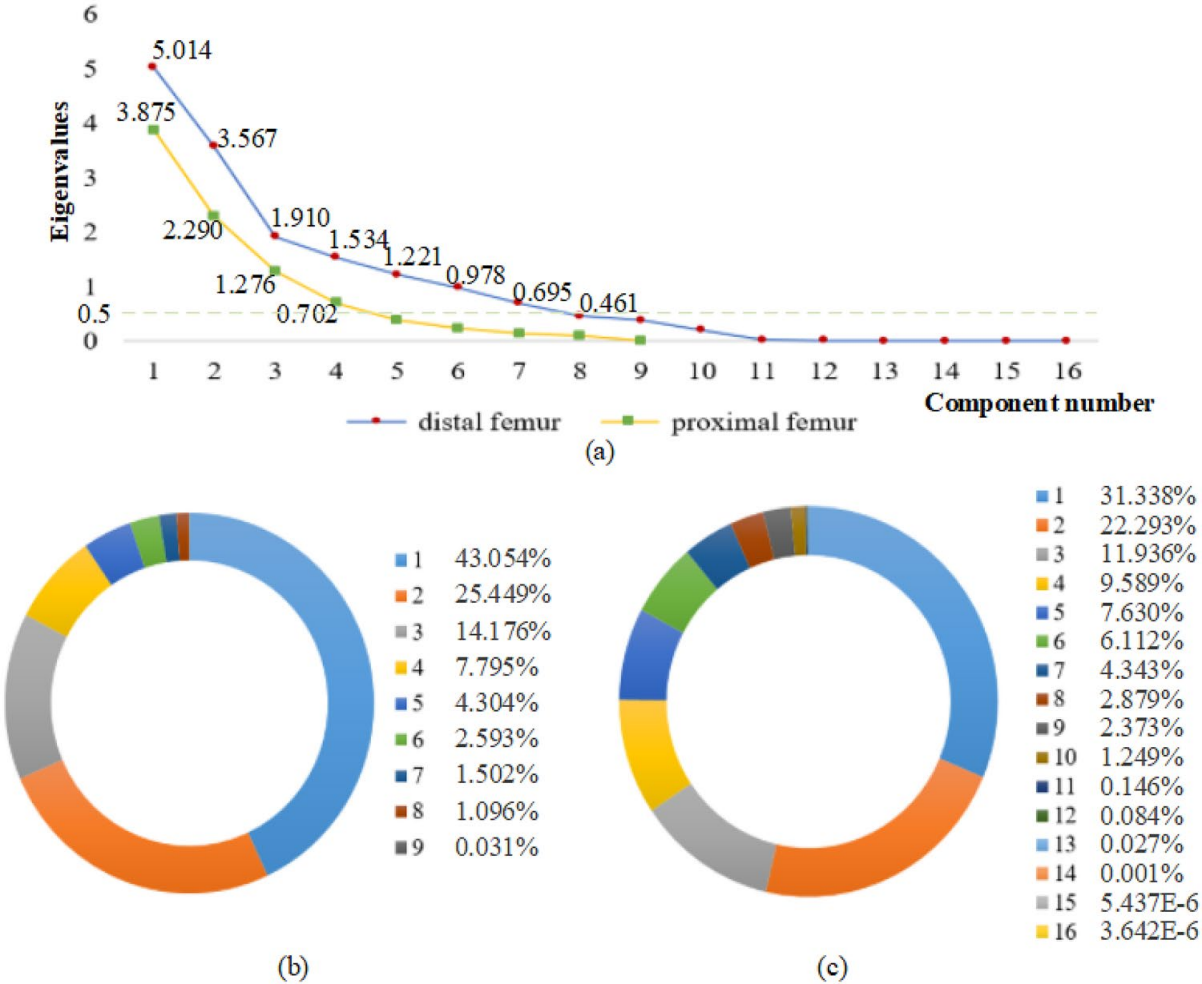
Components and their variance contribution rate: (**a**) components and their corresponding eigenvalues, (**b**) variance contribution rate of the components of the proximal femur, and (**c**) variance contribution rate of the components of the distal femur.

1. For the proximal femur, the yellow line in Fig. 2a indicates the curve change of the eigenvalue with component number. Notably, the first eigenvalue is the largest and those after the fifth are smaller and only change slightly. Therefore, we selected the first four components. Table 1 lists the principal component load matrix of the proximal femur, *A*_ij_ (each element in the matrix is denoted *a*_*ij*_, the range of *i* is 1–4, and range of *j* is 1–9). The column of each principal component constitutes the eigenvector corresponding to the principal component. For example, the eigenvectors corresponding to principal component 1 were 0.348, − 0.070, 0.223, 0.243, 0.466, 0.446, 0.450, 0.275, and 0.269, respectively. Equation (1) shows the mathematical model of the PCA of proximal femoral parameters, i.e., the correlation between the principal components, *p*_1_, *p*_2_, *p*_3_, and *p*_4_, and the original variables, *X*_1_ – *X*_9_.
  1 $$p_{i} = \sum\limits_{j = 1}^{9} {a_{ij} X_{j} } \begin{array}{*{20}c} {} & {(i = 1,2,3,4)} \\ \end{array}$$
2. For the distal femur, the blue line in Fig. 2a indicates a change in the eigenvalue with component number. The curve becomes less steep from the eighth component onward. Therefore, we selected the first seven components. Table 2 lists the principal component load matrix of the distal femur, *B*_ij_, where each element in the matrix is called *b*_*ij*_, the range of *i* is 1–7, and the range of *j* is 1–16. The column of each principal component constitutes the eigenvector corresponding to the principal component. Equation (2) shows the mathematical model of PCA, i.e., the correlation between the principal components, *q*_1_, *q*_2_, *q*_3_, *q*_4_, *q*_5_, *q*_6_, and *q*_7_, and the original variables, *Y*_1_–*Y*_16_.
  2 $$q_{i} = \sum\limits_{j = 1}^{16} {b_{ij} Y_{j} } \begin{array}{*{20}c} {} & {(i = 1,2,3,...,7)} \\ \end{array}$$

**Table 1:** Principal component load matrix of the proximal femur.

*a*_*ij*_		*i*			
		1	2	3	4
*j*	1	0.348	0.291	0.384	0.289
	2	− 0.070	0.448	0.606	0.137
	3	0.223	− 0.571	− 0.058	0.283
	4	0.243	− 0.441	0.353	0.457
	5	0.466	0.058	− 0.010	− 0.178
	6	0.446	0.137	− 0.140	− 0.156
	7	0.450	0.108	− 0.177	− 0.004
	8	0.275	0.326	− 0.421	0.273
	9	0.269	− 0.233	0.359	− 0.691

**Table 2:** Principal component load matrix of the distal femur.

*b*_*ij*_		*i*						
		1	2	3	4	5	6	7
*j*	1	− 0.020	− 0.065	− 0.069	− 0.164	0.157	0.951	0.944
	2	0.425	− 0.033	− 0.185	0.092	− 0.094	0.022	0.866
	3	0.425	− 0.033	− 0.185	0.092	− 0.094	0.021	0.866
	4	0.230	− 0.244	0.227	− 0.230	0.431	− 0.140	0.988
	5	0.268	− 0.032	0.357	− 0.207	0.384	− 0.101	0.984
	6	0.420	− 0.068	− 0.198	0.086	− 0.095	0.018	0.867
	7	0.427	− 0.013	− 0.174	0.096	− 0.094	0.022	0.862
	8	− 0.059	− 0.486	− 0.212	− 0.101	− 0.043	− 0.053	0.861
	9	0.040	0.489	0.207	0.080	0.020	0.016	0.794
	10	− 0.042	− 0.138	0.171	0.581	0.279	0.053	0.668
	11	− 0.007	− 0.142	0.064	0.607	0.309	0.105	0.819
	12	0.001	0.106	− 0.472	− 0.159	0.606	− 0.166	0.747
	13	0.162	− 0.254	0.535	− 0.041	− 0.192	0.019	0.979
	14	0.278	0.393	0.082	0.124	− 0.037	0.043	0.812
	15	− 0.202	0.115	− 0.182	0.251	− 0.011	− 0.106	1.588
	16	0.062	0.415	0.090	− 0.128	0.163	0.079	0.905

**Figure 3:**
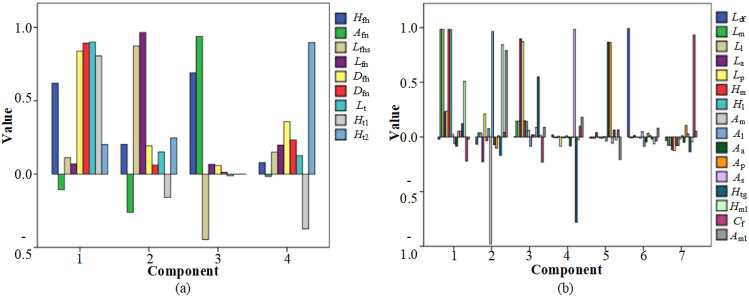
Rotated components of (**a**) proximal parameters and (**b**) distal parameters.

In this experiment, as shown in Fig. 2b, the cumulative variance contribution rate^31^ of the proximal femur was 90.474%, whereas the variance contribution rates of the first four components were 43.054%, 25.449%, 14.176%, and 7.795%, respectively. As shown in Fig. 2c, the cumulative variance contribution rate of the distal femur was 93.241%, whereas the variance contribution rates of the first seven components were 31.338%, 22.293%, 11.936%, 9.589%, 7.630%, 6.112%, and 4.343%, respectively. The extracted principal components can explain the original data well. In addition, to interpret the principal component representative of the morphological parameters, we adopted the maximum variance orthogonal rotation method^31^. Figure 3 shows the rotated principal components of the proximal and distal parameters. In Fig. 3a, Component 1 primarily explains *D*_fh_, *D*_fn_, *L*_t_, and *H*_t1_; Component 2, *L*_fhs_ and *L*_fn_; Component 3, *H*_fh_ and *A*_fn_; and Component 4, *H*_t2_. In Fig. 3b, Component 1 primarily explains *L*_f_, *L*_l_, *H*_m_, and *H*_l_; Component 2, *A*_m_ and *A*_l_; Component 3, *L*_a_ and *L*_p_; Component 4, *A*_s_ and *H*_tg_; Component 5, *A*_a_ and *A*_p_; Component 6, *L*_df_; and, finally, Component 7, *C*_f_.

We further calculated the weighted sum of the extracted principal components to obtain the final evaluation value, which is the variance contribution rate of each principal component. We divided the femur samples into three categories based on the principal component composite scores. We used variance analysis to verify the rationality of the classification. The results confirmed that most morphological parameters were statistically significant in the differences between classes (i.e., Sig. less than 0.05), indicating classification rationality. This suggests that the extraction method used in this study is reasonable and effective.

## Construction of the plate feature model

Our previous work was based on bone plate features based on the idea of features^32^. Plate features were formalized into a quadruple form, including constraint relationships, mapping relationships, and semantic parameters. Because its shape contained a feature point, feature curve, and surface, a plate was expressed at a higher level to avoid the tedious process of designing from the bottom; this improves the design efficiency and quality. By following the same rationale for this study, we enhanced the plate feature model by integrating the bone’s morphological features to set the semantic parameters of the plate’s features. The key steps included the representation of the relation, semantic feature parameters, and mapping relation.

### Constraint relationships

**Figure 4:**
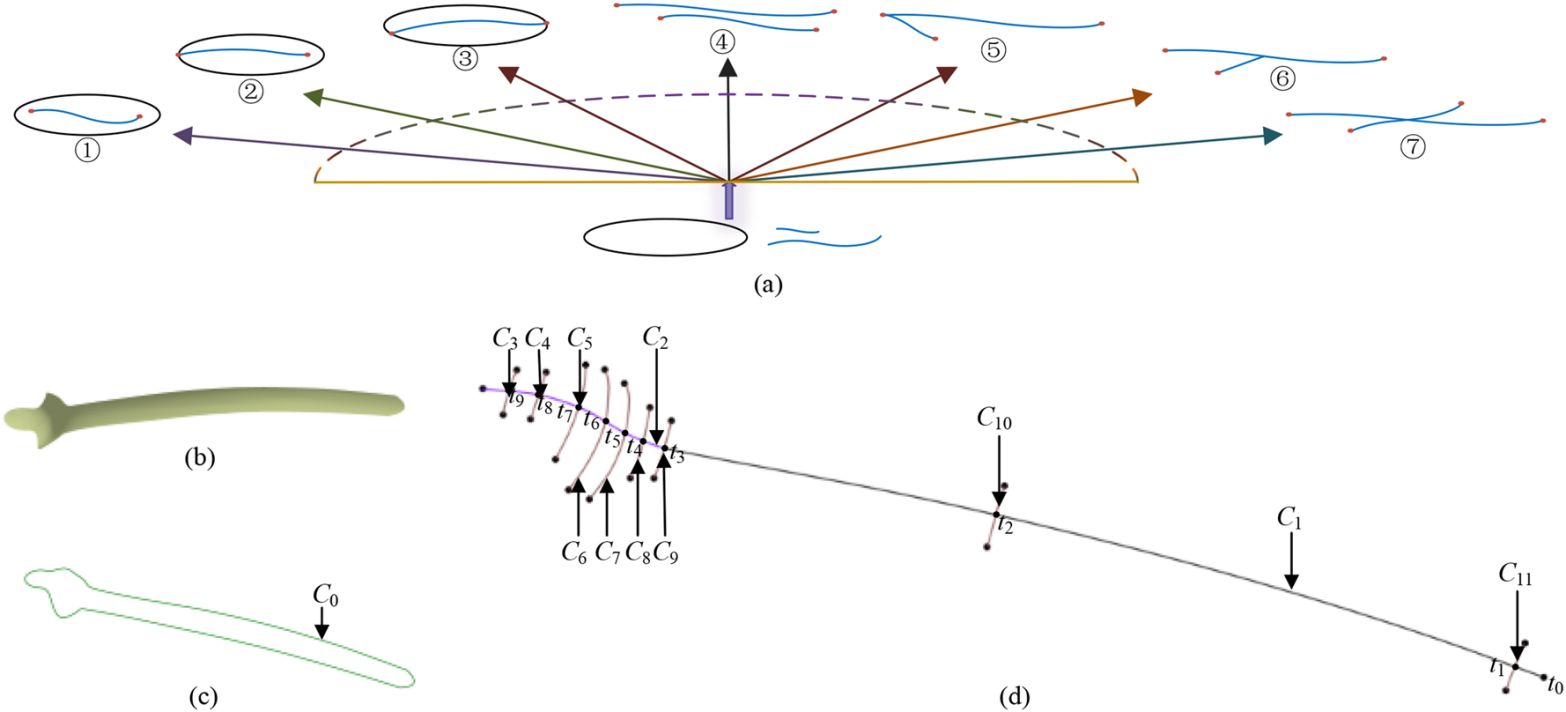
Skeleton-like structure of the undersurface of the eagle-shaped plate: (**a**) topological relations between feature curves: ① *BI*_0 type, ② *BI*_11 type, ③ *BI*_21 type, ④ *II*_0 type, ⑤ *II*_11 type, ⑥ *II*_12 type, and ⑦ *II*_13 type. (**b**) undersurface, (**c**) boundary feature curve, and (**d**) ridge curves and costal curves.

The constraint relation of the surface features primarily includes structural and dimensional constraints^33–35^. Typically, shape features represent the plates. The structural constraint is also referred to as the shape constraint. The shape contains points, curves, and surfaces. The curve is an important bridge from a point to a surface and its topological relation determines the feature contour shape of the surface. As a result, shape constraints are mainly reflected in the topological relationships between the feature curves. For the undersurface of the orthopedic plates, the feature curves primarily include the boundary curve (referred to as B) and internal auxiliary curve (referred to as I). For brevity, the numbers represent the position (separation or intersection) between the two feature curves, as shown in Fig. 4a. The topological relation between the boundary feature curve and the internal auxiliary feature curve is called the *BI* relation, based on the number of intersection points between the boundary feature curve and the internal auxiliary feature curve. The topological relation between any two internal auxiliary feature curves, called the II relation, is based on the position of the intersection points between the two internal auxiliary feature curves.

Figure 4b–d shows the undersurface of the eagle-shaped plate constructed as a skeleton structure. The bone plate has a good mechanical effect^36^ and is accepted as fracture types 31A3 and 32A3^37^. The feature curves contain the boundary feature curve (*C*_0_) and the auxiliary feature curves. We used a boundary feature curve to represent the edge profiles. The auxiliary feature curve also resembles the skeleton and contains ridge curves (*C*_1_ and *C*_2_) and costal curves (from *C*_3_ to *C*_11_). The following adjacency matrix represents the structural constraints of the feature curves.

$$\left[ {\begin{array}{*{20}c} B & {BI\_11} & {BI\_11} & {BI\_21} & {BI\_21} & {BI\_21} & {BI\_21} & {BI\_21} & {BI\_21} & {BI\_21} & {BI\_21} & {BI\_21} \\ {} & I & {II\_11} & {II\_0} & {II\_0} & {II\_0} & {II\_0} & {II\_0} & {II\_0} & {II\_12} & {II\_13} & {II\_13} \\ {} & {} & I & {II\_13} & {II\_13} & {II\_13} & {II\_13} & {II\_13} & {II\_13} & {II\_12} & {II\_0} & {II\_0} \\ {} & {} & {} & I & {II\_0} & {II\_0} & {II\_0} & {II\_0} & {II\_0} & {II\_0} & {II\_0} & {II\_0} \\ {} & {} & {} & {} & I & {II\_0} & {II\_0} & {II\_0} & {II\_0} & {II\_0} & {II\_0} & {II\_0} \\ {} & {} & {} & {} & {} & I & {II\_0} & {II\_0} & {II\_0} & {II\_0} & {II\_0} & {II\_0} \\ {} & {} & {} & {} & {} & {} & I & {II\_0} & {II\_0} & {II\_0} & {II\_0} & {II\_0} \\ {} & {} & {} & {} & {} & {} & {} & I & {II\_0} & {II\_0} & {II\_0} & {II\_0} \\ {} & {} & {} & {} & {} & {} & {} & {} & I & {II\_0} & {II\_0} & {II\_0} \\ {} & {} & {} & {} & {} & {} & {} & {} & {} & I & {II\_0} & {II\_0} \\ {} & {} & {} & {} & {} & {} & {} & {} & {} & {} & I & {II\_0} \\ {} & {} & {} & {} & {} & {} & {} & {} & {} & {} & {} & I \\ \end{array} } \right]$$

The dimension constraint mainly restricts the semantic parameters and contains two meanings: the correlation between the semantic parameters and the value range of each semantic parameter. These semantic parameter relations and range values are based on the relationship between the bone morphological parameters and the range of values.

### Semantic feature parameters

Size parameters include global feature parameters that reflect the common shape of the plate and local parameters, which are constructed by dividing the global parameters into several special feature parameters according to different angles. Furthermore, we constructed several generic feature parameters using the attributes of the global parameters. We also constructed several special feature parameters using the local parameter attributes. The semantic feature parameters primarily refer to those with certain semantics that support high-level operations^38^. The definition of feature parameters corresponds to the specific engineering semantics and functional information, facilitating surface feature instantiation.

**Figure 5:**
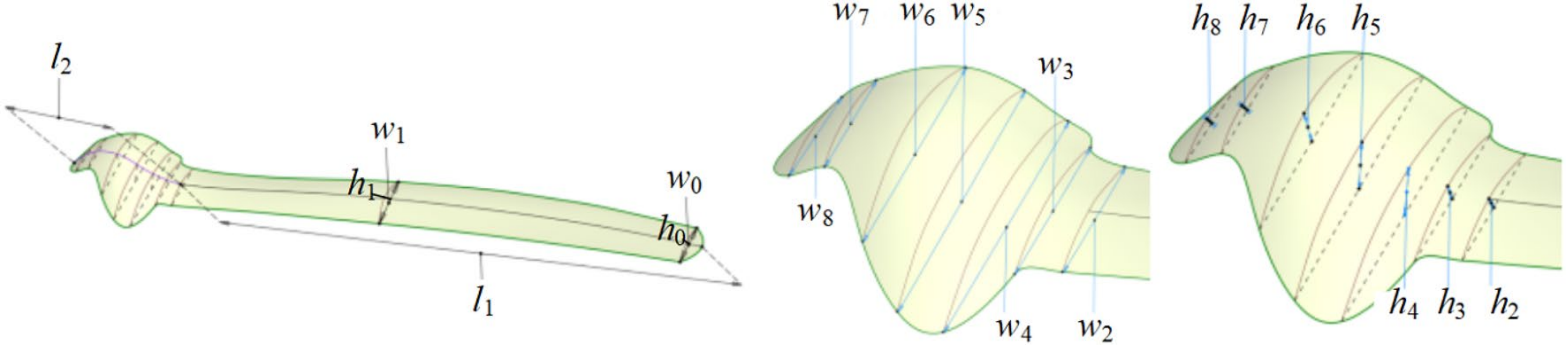
Size parameters of the undersurface of the eagle-shaped plates.

As shown in Fig. 5, the size parameters of the undersurface of the eagle-shaped plate include the total length *L*, total width *W*, tail length *l*_1_, head length *l*_2_, local tail widths *w*_0_ and *w*_1_, local head width *w*_i_ (*i* = 2, 3, …, 8), local tail bump height *h*_j_ (*j* = 0, 1), and local head bump height, *h*_j_ (*j* = 2, 3, …, 8). Moreover, *L* and *W* are mainly determined by the bone morphological parameters, including femur length *H*_f_ and femoral shaft coronal diameter *D*_fs_. *L* is divided into tail length and head length by inheritance; similarly, *W* is divided into tail width and head width by inheritance.

### Mapping relations

Based on the concept of layering from whole to part, the mapping relation is divided into two levels^38^:

- Primary level mapping refers to the correspondence between the feature parameters of the upper layer and the feature curves of the middle layer.
- Secondary level mapping refers to the correspondence between the feature points of the middle and bottom layers.

**Figure 6:**
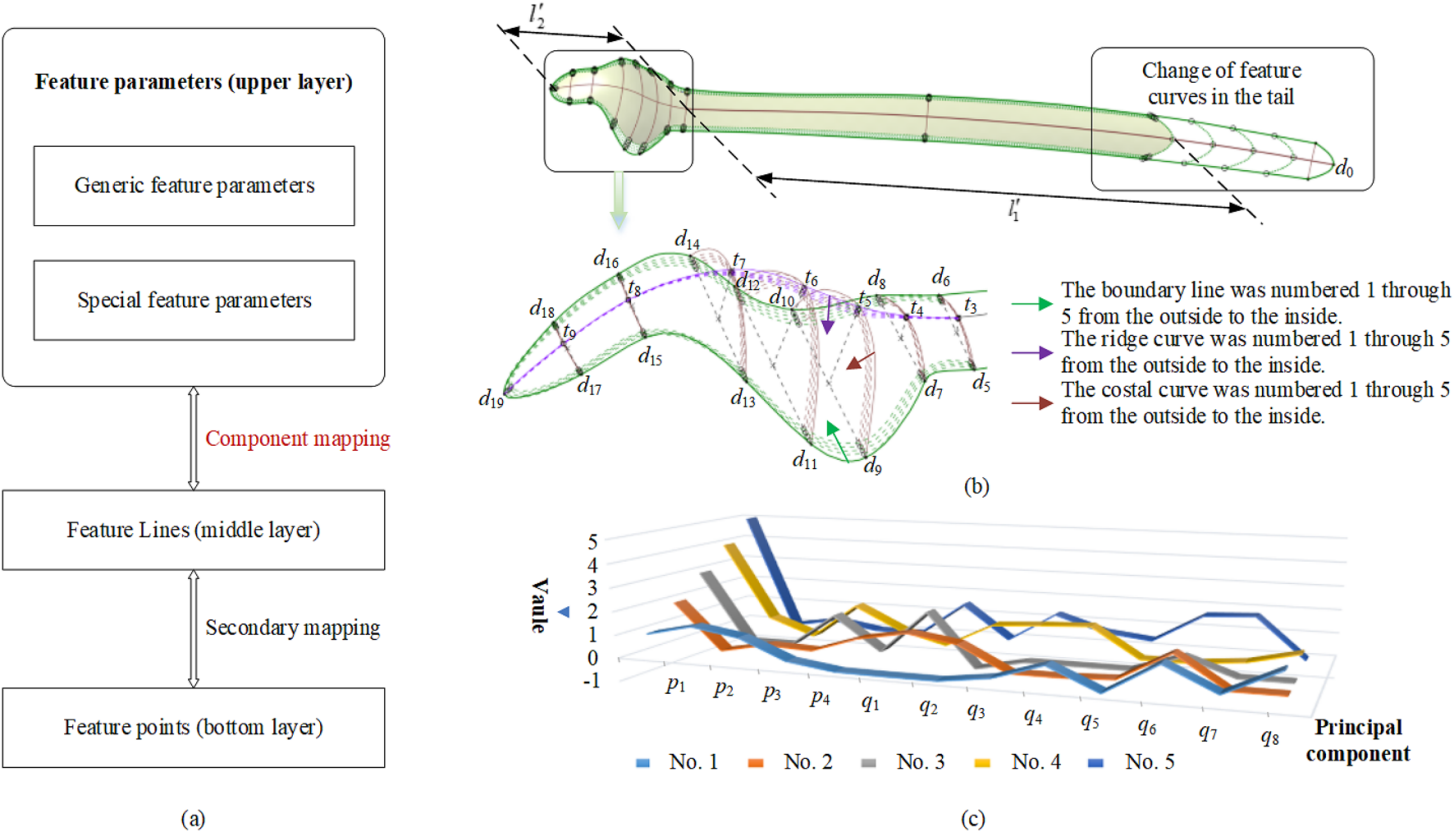
Undersurface feature change of the eagle-shaped plate. (**a**) Mapping relation of the feature model. The upper layer containing the feature parameters can be constructed based on the bone information. Through the component mapping and secondary mapping, the correspondence between the feature parameters and the underlying element is established, allowing the feature parameters to uniquely determine the feature geometric shape, (**b**) change of feature curves. The tail and head parameters change based on different principal component values. The feature curve approaches inward under the constraints of boundary and internal auxiliary feature curves, and (**c**) principal component values of numbered curves 1–5 shown in (**b**).

Because this mapping relationship did not adequately incorporate bone morphology, the constructed plate feature was relatively independent and, as a result, did not fit well with the bone morphological features. In this study, based on the above research, we integrated the principal component into the primary mapping to form a new mapping relation, which is called component mapping (see Fig. 6a). The mapping relation is represented as $$\begin{gathered} F_{{1}} = {{\{ X}} \to {\text{Y|X}} \in {\text{P}}_{1} {\text{,Y}} \in {\text{P}}_{2} \} \hfill \\ F_{{2}} = {{\{ Y}} \to Z{\text{|Y}} \in {\text{P}}_{2} {\text{,Z}} \in {\text{P}}_{3} \} \hfill \\ \end{gathered}$$, where *F*_1_ is the primary mapping, *F*_2_ is the secondary mapping, *P*_1_ denotes the upper layer, *P*_2_ denotes the middle layer, and *P*_3_ denotes the bottom layer. The component mapping, $F_{{1}}^{\prime }$, is $F_{{1}}^{\prime } = \{ X\mathop{\longrightarrow}\limits^{{P_{{4}} }}{\text{Y|X}} \in {\text{P}}_{1} {\text{,Y}} \in {\text{P}}_{2} \}$, where *P*_4_ is the set of principal components, $P_{4} = \{p_{1} ,p_{2} ,p_{3} ,p_{4} ,q_{1} ,q_{2} ,q_{3} ,q_{4} ,q_{5} ,q_{6} ,q_{7} \}$, extracted as described in “Extraction of bone morphological features” section. Here, we add the principal components to the mapping as weights. Figure 6b illustrates this, and Fig. 6c shows the principal component values.

## Generation of the customized plate

**Figure 7:**
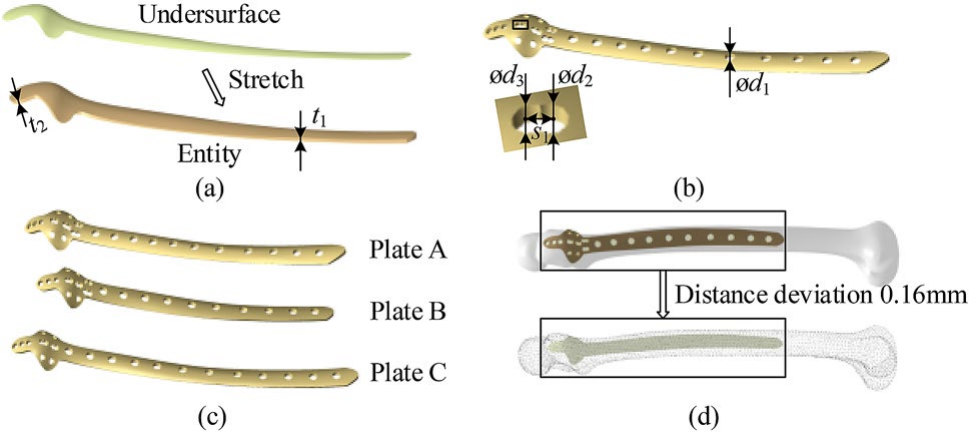
Parameter variation of the features in the eagle-shaped plate: (**a**) entity generated from undersurface, (**b**) punch holes and set parameters, (**c**) plates with different parameters. **Plate A**: *l*_1_ = 229 mm; *l*_2_ = 39 mm; *w*_0_ = 12 mm; *w*_1_ = 14 mm; *w*_2_ = 16 mm; *w*_5_ = 29 mm; *w*_7_ = 13 mm; *h*_2_ = 1.6 mm; *h*_3_ = 2.4 mm; *h*_4_ = 7.9 mm; *h*_5_ = 8.3 mm; *h*_2_ = 5.2 mm; *h*_7_ = 0.8 mm; *h*_8_ = 0.7 mm; *t*_1_ = *t*_2_ = 3 mm; ø*d*_1_ = 7 mm; ø*d*_2_ = ø*d*_3_ = 5 mm; and *s*_1_ = 6 mm. **Plate B**: *l*_1_ = 204 mm; *l*_2_ = 44 mm; *w*_0_ = 12 mm; *w*_1_ = 14 mm; *w*_2_ = 15 mm; *w*_5_ = 25 mm; *w*_7_ = 13 mm; *h*_2_ = 1.5 mm; *h*_3_ = 2.2 mm; *h*_4_ = 7.2 mm; *h*_5_ = 8.6 mm; *h*_2_ = 5.3 mm; *h*_7_ = 1.0 mm; *h*_8_ = 0.9 mm; *t*_1_ = *t*_2_ = 3 mm; ø*d*_1_ = 7 mm; ø*d*_2_ = ø*d*_3_ = 5 mm; and *s*_1_ = 6 mm. **Plate C**: *l*_1_ = 248 mm; *l*_2_ = 40 mm; *w*_0_ = 12 mm; *w*_1_ = 14 mm; *w*_2_ = 17 mm; *w*_5_ = 33 mm; *w*_7_ = 14 mm; *h*_2_ = 1.4 mm; *h*_3_ = 2.8 mm; *h*_4_ = 7.3 mm; *h*_5_ = 8.0 mm; *h*_2_ = 4.7 mm; *h*_7_ = 1.2 mm; *h*_8_ = 1.3 mm; *t*_1_ = *t*_2_ = 3 mm; ø*d*_1_ = 7 mm; ø*d*_2_ = ø*d*_3_ = 5 mm; and *s*_1_ = 6 mm. and (**d**) fit effect of the eagle-shaped plate to femur. CATIA URL: https://www.3ds.com/zh/products-services/catia/.

We edited the semantic parameters to generate customized features of the eagle-shaped plate using Microsoft Visual C++ 2008 and *Dassault Systèmes CATIA V5R22*. We modified tail length *l*_1_ by changing the feature points of the tail. We also modified the local width *w*_i_ (*i* = 1, 2, 3, …, 8) and height *h*_i_ (*i* = 1, 2, 3, …, 8) by changing the boundary feature points in the head. Figure 7a shows how the entity of the eagle-shaped plate was generated by stretching the undersurface; Fig. 7b shows that the entity parameters include the tail thickness *t*_1_, head thickness *t*_2_, and hole parameters (ø*d*_1_, ø*d*_2_, ø*d*_3_, and *s*_1_); Fig. 7c shows the eagle-shaped plates with different parameters.

Figure 7d shows good adhesion between the designed eagle-shaped plate and femur. To prove this further, we calculated the distances between the feature points on the femoral contact area and the undersurface of the plate. The results show that every distance deviation was controlled within 0.16 mm; this shows that the plate designed in this study matches well with the bone and thus guarantees the design requirement of the bone plate. Table 3 presents the comparison results between the proposed method and existing design methods. Notably, this method constructs a customized bone plate by blending bone morphological features into the semantic layer of the plate feature model; thus, design efficiency can be improved by avoiding the design from scratch while ensuring a match between the bone plate and the bone.

**Table 3:** Comparison between the proposed method and existing methods.

Compare item	Soni^12^	Liu^13^	Chen ^14^	He^15^	This method
Bone feature extracted?	No	Yes	Yes	No	Yes
Parameters mapping relation established?	No	No	Yes	Yes	Yes
Parameterization of plate	No	No	Yes	Yes	Yes
Operation	General	Well	Well	Well	Well
Re-design of the features	General	Simple	Simple	Simple	Simpler

## Discussion and conclusion

Conventional design methods for customized plates heavily rely on interactions with medical professionals. They also have a low level of reusability and often result in large deviations from the design objectives. In this study, we extracted bone morphological features using the PCA method and incorporated them as weights into the mapping relation to construct plate feature models. The main contributions of this study are as follows:

- First, to the best of our knowledge, this is the first study in which the bone’s morphological features and plate features are fully encapsulated with an internal correlation mechanism. Undoubtedly, this greatly improved the matching degree between the plate and bone. The construction of the feature parameter set of the plate inherits the existing features and thus avoids unnecessary duplication of features.
- For the second contribution, we obtained different plate features by adjusting the upper-level feature parameters. This improves the flexibility and applicability of the design, reducing the repetitive tasks required in conventional methods and shortening the plate design cycle.

The experimental results show that the constructed plate feature model is competitive in design convenience because it facilitates quick and efficient intuitive editing of the feature parameters. The proposed method can be easily extended to other types of bones and plates and can provide insights, methods, and techniques for other fields of customized design.

Notably, we did not introduce the feature parameter modification sequence of the plate feature model in this study. To address this potential disadvantage, future studies should prioritize setting the feature parameters. Further consideration should also be given to the hardness parameters of the bone plate and appropriate materials should be reasonably selected. More exciting research can be conducted on a feature template that can be constructed by integrating a group of bone morphological features with the same fracture type and different anatomical morphology into a set of semantic feature parameters of the plate object.
